# Atlanta Medical College

**Published:** 1856-03

**Authors:** 


### EDITORIAL AND MISCELLANEOUS.

### ATLANTA MEDICAL COLLEGE.

We again recur to the subject of this Institution, for the purpose of setting forth, briefly, its present position and future prospects, and a declaration of the principles by which the Trustees and Faculty will be guided—demanded, as we conceive, by the circumstances which surround us.

Commencing under the most unfavorable auspices, apparently, for its success, it has continued to progress in spite of every obstacle, naturally existing in the way of such an enterprise, combined with the assaults of enemies, both open and covert.

Emerging, again and again, from what seemed to be the dark cloud of destruction, with more and more strength, gathered from the contest, it has succeeded in winning the confidence, not only of the community in which it is located, but of a large and influential number of professional men, and others, throughout our own State, as well as to an extent in other States, that justifies us, we think fully, in asserting, that but few institutions, medical or others, have attained an equal position at the same age.

We say thus much in no boasting spirit, but rather mention it as calling for gratitude to an overruling Providence (strikingly illustrated, as we are sometimes disposed to think, in the results of a number of apparently discouraging circumstances connected with the history of this movement,) and to the many kind friends who have manifested an interest in the enterprise, and who have freely given their aid to its success.

While the above is true, and the Atlanta Medical College, in all essential particulars, is now as fully established as any one of the oldest of our medical institutions—regularly chartered by a sovereign State, with all powers and privileges requisite for the full performance of all the functions belonging to any medical school, and able to exhibit a list of students and graduates, comparing favorably, with those of the most prominent and successful colleges in their commencement—it is at the same time, also, true that we have yet a great work to perform, and are feeling fully the importance and necessity of continued and energetic action: and can assure those who are feeling an interest in the matter, that the individuals who conceived the plan, and brought the energy, which has resulted in its present firm and advancing position, are not idle, nor resting upon the results achieved by previous labors, but with every thing to encourage confidence in the final success of the enterprise, are concentrating every power to complete the buildings, and furnish the necessary apparatus, at the earliest possible day.

Owing, however, to the great pecuniary pressure of the times, and the consequent inability to command the requisite funds, and the failure upon the part of the Legislature to make an appropriation which we had a right to expect, in addition to the excessively severe winter, (completely arresting our building operations,) it is now probable that we shall not be able to occupy the college buildings at the commencement of the course of lectures, though possibly, at some time during the session, if it should be thought desirable.

But however anxious the Trustees and Faculty may be to complete the buildings, they are not willing for any mere temporary advantage, which might be supposed to flow from a speedy occupancy of the college edifice proper, to forego what they consider of far more importance—the faithful execution of a permanent structure, and one that will meet the demands of the prosperous institution, now clearly foreshadowed, and that will be creditable to the City and State.

But we can assure those who are designing to spend the next session with us, that the non-fulfillment of our anticipations in this particular, will not, in the slightest degree, interfere with an entire preparation for the accommodation and teaching of all who may come; the lecture rooms will be spacious and well adapted to the purpose, and in every respect, our facilities for instruction will greatly exceed those of the first course.

And in this connection, we would remark, that the projectors of this enterprise are not under the influence of any such selfish and contracted view, as to have for their object, merely to build up a patronage, and to vie with rival schools for *num-*

*bers*, but with a comprehensive sense of the obligations resting upon them, as the representatives of a science, devoted to the elucidation of the most important truths connected with our temporal existence, are laboring to lay a broad and solid foundation, with an enduring superstructure, that will, in a higher and more important sense, compete successfully with the first institutions in the land; and if there are any individuals or institutions belonging to the profession, whose course indicates, that in their estimation, "they are the people, and wisdom will die with them"—and who are setting themselves up as the general regulators of medical affairs, and are disposed to dictate what we shall, or shall not, do, we would say to them, that while we are perfectly satisfied with our position, (and the ample recognition of our claims, upon the part of other medical colleges,) as being fully endowed with rights and powers, fully equal to those emanating from any government in this or any other country, and therefore, if preferred, perfectly independent of any *self-constituted* tribunal whatever, we still choose to place ourselves upon the same platform with the other representatives of true and regular medicine, and wish it to be distinctly understood, that our intention is to have a standard not inferior, in any particular, to that which shall be adopted and sustained by the voice and action of the profession, and in whatever matter, reform or improvement, may be demanded, we shall be prepared to come up to the full requisition, and shall, at all times be found as active and zealous coöperators for the more thorough education of medical men—whether it be by more rigid requirements in reference to preliminary education, a higher standard to regulate examinations, the separation of the teaching and licensing power, or a prolonged term of study; and in reference to the latter, we do not mean those catchpenny and pseudo extensions of terms, denominated preliminary lectures, whose object is now too well understood to make it necessary to do more than barely allude to them, but a *bona fide* establishment of some uniform increase in the time of study and attendance upon lectures, before an individual shall be allowed to become a candidate for the degree.

As to the position we have been disposed to occupy in reference to our relations, with others engaged in the same work, it has been (in our opening exercises) already fully announced in terms befitting the occasion, and we have only to add, that, so far as any public and open manifestation of feeling and sentiment has been exhibited, we are not disposed to complain.

It is true, that to a *very small* extent, there has been a show of arrogance and presumption, not perhaps, entirely agreeable, but not so formidable as to excite any serious apprehensions of entire extinction, or very surprising to those acquainted with the selfishness of fallen humanity.

We are, therefore, happy to have it to say, that the friendly and fraternal spirit which has been exhibited, from the first, upon the part of the founders of this institution, has been generally reciprocated; and upon the whole, that there has been a just and liberal acknowledgment of our rights, and a generous reception of our proffered services to aid in the great work of preparing young men for the medical profession.

From the information we are receiving from various quarters, our anticipations are sanguine as to the number who are designing to attend the next course of lectures here; being confident that we shall number in our class, students from some six or seven States, and are confirmed in the opinion expressed in our first issue, that our second class will double in number the first; but however large or elevated the character of any future class, they never can efface from our memory, the noble band of young men who devoted themselves with such untiring assiduity to the pursuit of their studies, and who held up the hands of the professors in so many ways, and whose conduct in every particular, was so unexceptionable, as to establish here, at least, an enviable reputation for the medical student; and in this connection, we will say in all candor, that we have very great doubts, whether it has ever occurred, that a stronger mutual attachment has arisen from similar relations between teachers and pupils, than that which we are certain, resulted from the association of the faculty and students during the first term; and in conclusion, and as testifying our respect and regard—and wishing them that full success in life, which their devotion to duty, while here, has given us a right to expect—we dedicate our article to the "First Class in the Atlanta Medical College."

